# Assessment of the effectiveness of three different cephalosporin/clavulanate combinations for the phenotypic confirmation of extended-spectrum beta-lactamase producing bacteria isolated from urine samples at National Public Health Laboratory, Kathmandu, Nepal

**DOI:** 10.1186/s13104-016-2192-2

**Published:** 2016-08-04

**Authors:** Raju Bhandari, Narayan Dutt Pant, Asia Poudel, Mukunda Sharma

**Affiliations:** 1 Department of Microbiology, Goldengate International College, Battisputali, Kathmandu, Nepal; 2 Department of Microbiology, Grande International Hospital, Dhapasi, Kathmandu, Nepal; 3 National Public Health Laboratory, Teku, Kathmandu, Nepal

**Keywords:** Extended-spectrum beta-lactamases, Uropathogens, Antibiotic susceptibility patterns, Nepal

## Abstract

**Background:**

The extended-spectrum β-lactamase (ESBL) producing bacteria are present as the serious public health problems due to their resistance to large number of antibiotics. The main aims of this study were to determine the prevalence and antibiotic resistance patterns of bacteria producing extended-spectrum β-lactamases (ESBLs) and to find the suitable cephalosporin/clavulanate combination for phenotypic confirmation of ESBL production.

**Methods:**

During the study period from April 2013 to November 2013, a total of 1003 urine samples from the patients visiting National Public Health Laboratory, Kathmandu, Nepal were collected and processed. The isolates were identified with the help of colony characteristics, gram stain and conventional biochemical tests. Antimicrobial susceptibility testing was performed by Kirby Bauer disc diffusion method. ESBL production screening was done by using ceftriaxone, while ESBL production confirmation was done by using three different 3rd generation cephalosporin/clavulanate combinations.

**Results:**

Of the 138 isolates, *Escherichia coli* was the most predominant with 88 (63.8 %) isolates. Among the antibiotics tested for gram negative bacteria, highest susceptibility was seen toward imipenem followed by amikacin. Of the total isolates, 68 (49.3 %) were suspected as ESBL producers. Of these, 44 (64.7 %) were phenotypically confirmed to be ESBL producers. The majority of ESBL producers were *E. coli* with 34 (72.3 %) isolates. Of the three different 3rd generation cephalosporin/clavulanate combinations used, ceftazidime/clavulanate combination was found to be most effective for phenotypic confirmation of ESBL producers and was statistically highly significant (P < 0.01).

**Conclusion:**

Based on the findings of our study, we recommend to use ceftazidime/clavulanate combination for phenotypic confirmation of ESBL producers. Routine ESBL testing for uropathogens along with conventional antibiogram would be useful for proper early management of all the cases of urinary tract infections.

## Background

Urinary tract infection (UTI) is one of the most common bacterial infections in humans both in the community and hospital setting, and are associated with significant morbidity and mortality as well as high economic burden on society [1]. The disease may be asymptomatic, but if symptoms develop they include cloudy or bloody urine, low-grade fever, back discomfort, fatigue, nausea and vomiting [2]. Most common causative agents of UTI are bacteria of enteric origin and among them *Escherichia coli* is the most frequent uropathogen [3]. Antibiotic resistance in bacteria is increasing worldwide in both out patients and hospitalized patients. It varies according to geographic location and the haphazard use of antibiotics is playing a significant role in the emergence of the drug resistant bacteria [4, 5]. The β-lactamases are the major defense of gram negative bacteria against β-lactam antibiotics. Extended-spectrum β-lactamase (ESBL) producing organisms have been increasingly detected worldwide with their varying prevalence in different settings and the extent of the problem is under-recognized due to unawareness and poor laboratory detection and reporting at many centers [6]. Considering, the rapid rise in ESBL producing organisms and the lack of accurate figures about the prevalence and the resistance patterns of clinically relevant bacteria producing ESBL in Nepalese population, we conducted this study. Further this study will be helpful for the laboratory personnel to choose most effective cephalosporin/clavulanate combination for phenotypic confirmation of ESBL production.

We processed urine samples of patients suspected of UTI to determine prevalence and the antibiotic susceptibility patterns of clinically relevant bacteria producing extended spectrum β-lactamases and to find the suitable cephalosporin/clavulanate combination for phenotypic confirmation of ESBL producing bacteria.

## Methods

The study was conducted at National Public Health Laboratory, Teku, Kathmandu, Nepal from April 2013 to November 2013. During this period, a total of 1003 urine samples from patients suspected of UTI were collected and processed by using standard laboratory methods.

### Sample collection and transportation

The patients were given a clean, wide mouth, dry and sterile leak proof container and requested for 5–10 ml mid-stream urine sample. Before providing the container, each patient was instructed properly for the collection of sample. The container was then labelled properly and immediately delivered to the laboratory with the request form for further processing. The quality of the sample was evaluated before processing in terms of proper labelling, visible signs of contamination, improper screwing of cap etc. The improper sample was rejected and the patient was requested to submit next sample.

### Culture of specimen

Culture of each urine sample was done on Cystine Lactose Electrolyte Deficient agar plate. An inoculating loop of standard dimension was used to take up fixed and a known volume (0.001 ml) of mixed uncentrifuged urine for inoculation. After inoculation the plates were incubated aerobically at 37 °C for 24 h and were observed for significant growth (10^5^ cfu/ml).

### Identification of the isolates

Isolated bacteria were identified by using standard microbiological tools and techniques as described in the Bergey’s manual of systematic bacteriology which involve morphological appearance of the colonies, staining reaction and biochemical properties. Each of the organism was isolated in pure form before performing biochemical tests. The biochemical tests used for the identification included catalase test, oxidase test, sulfide indole and motility test, methyl red test, Voges Proskauer test, citrate utilization test, oxidation fermentation test and triple sugar iron test.

### Antibiotic susceptibility testing

The antimicrobial susceptibility testing was done by modified Kirby-Bauer disk diffusion method as recommended by Clinical and Laboratory Standards Institute (CLSI) using Mueller–Hinton agar [7].

### Screening and confirmation of ESBL producing isolates

Bacterial isolates were first tested with ceftriaxone (30 µg) as per the CLSI screening criteria. The isolates were suspected to be ESBL producers, if zone of inhibition was ≤25 mm. The suspected ESBL producing isolates were tested for confirmation for ESBL production by combined disc method, using cefpodoxime (10 µg) and cefpodoxime (10 µg) + clavulanate (1 µg), cefotaxime (30 µg) and cefotaxime (30 µg) + clavulanate (10 µg) and ceftazidime (30 µg) and ceftazidime (30 µg) + clavulanate (10 µg). An increase in zone diameter of ≥5 mm in the presence of clavulanate from any or all of the set was confirmed as ESBL production. The antibiotic discs used in the study were supplied by Hi-media laboratories pvt. ltd., India. As controls, *E. coli* ATCC 25922 (ESBL negative) and *Klebsiella pneumoniae* ATCC 700603 (ESBL positive) were used.

### Data analysis

SPSS version 19.0 was used for statistical analysis. Chi-square test was applied and P < 0.01 was considered statistically significant.

## Results

A total of 1003 urine samples were processed for culture, of which 611 (60.9 %) were from male patients and 392 (39.1 %) were from female patients. Of the 611 samples from male, 63 (10.3 %) showed significant growth. Similarly, of the 392 samples from female, 75 (19.1 %) showed significant growth.

### Distribution of urinary isolates

Total of 138 bacteria belonging to 13 different species were isolated (Table 1). Among the isolates gram negative bacteria were found predominant constituting 134  (97.10 %) out of 138. Of all the isolates, *E. coli* was the most frequently isolated species (63.8 %) followed by *Pseudomonas aeruginosa* (8.7 %). The least common isolates were *Edwardsiella* spp.*, Providencia* spp., and *Serratia* spp. Similarly gram positive bacteria constitute 2.9 % of the total isolates and *Staphylococcus aureus* was the only gram positive bacterium isolated.

**Table 1:** Gender wise distribution of urinary isolates

Organisms isolated	Male		Female		Total	
	No. of isolates	%	No. of isolates	%	No. of isolates	%
*Gram negative bacteria*						
*Acinetobacter* spp.	5	83.3	1	16.7	6	4.3
*Citrobacter koseri*	3	50	3	50	6	4.3
*Citrobacter freundii*	0	0	4	100	4	2.9
*E. coli*	38	43.2	50	56.8	88	63.8
*Edwardsiella* spp.	1	100	0	0	1	0.7
*Enterobacter* spp.	1	50	1	50	2	1.4
*Kl. pneumoniae*	2	25	6	75	8	5.8
*Morganella morganii*	1	50	1	50	2	1.4
*Proteus* spp.	2	66.7	1	33.3	3	2.2
*Providencia* spp.	1	100	0	0	1	0.7
*Pseudomonas aeruginosa*	8	66.7	4	33.3	12	8.7
*Serratia* spp.	0	0	1	100	1	0.7
*Gram positive bacteria*						
*S. aureus*	1	25	3	75	4	2.9
Total	63	45.7	75	54.3	138	100

Among the 138 growth positive samples, 63 (45.7 %) were from males and 75 (54.3 %) were from females. Among the different isolates, *E. coli* was the most predominant bacteria both in male 38 (43.2 %) and female 50 (56.8 %).

### Antibiotic susceptibility patterns of gram negative isolates

Out of 13 antibiotics tested for gram negative isolates, 7 different antibiotics (belonging to 5 different classes) were used as first line drugs and other six antibiotics were used as second line drugs. Among the first line drugs used, highest rate of susceptibility was shown to nitrofurantoin (61.9 %) and which is statistically significant (P < 0.01). Among the second line drugs used, highest rate of susceptibility was shown toward imipenem (96.4 %) followed by amikacin (91.1 %) and piperacillin/tazobactam (69.6 %). Ampicillin was found as the drug to which most strains were resistant with only 24.6 % of isolates being susceptible (Table 2).

**Table 2:** Antibiotic susceptibility patterns of gram negative isolates

Antibiotics used	Susceptible		Intermediate		Resistant		Total
	No	%	No	%	No	%	
*First line drugs*							
Ampicillin	33	24.6	0	0	101	75.4	134
Amoxycillin + clavulanic acid	49	36.6	2	1.5	83	61.9	134
Ciprofloxacin	57	42.5	1	0.7	76	56.7	134
Cotrimoxazole	51	38.1	0	0	83	61.9	134
Ceftriaxone	65	48.5	2	1.5	67	50	134
Nitrofurantoin	83	61.9	7	5.2	44	32.8	134
Norfloxacin	55	41	0	0	79	59	134
*Second line drugs*							
Amikacin	51	91.1	1	1.8	4	7.1	56
Cefoxitin	19	33.9	5	8.9	32	57.1	56
Ceftazidime	15	25.9	1	1.7	42	72.4	58
Gentamicin	26	46.4	2	3.6	28	50	56
Imipenem	54	96.4	0	0	2	3.6	56
Piperacillin/tazobactam	39	69.6	3	5.4	14	25	56

### ESBL production among various isolates

Of the 68 suspected ESBL positive isolates, 44 (64.7 %) were ESBL positive whereas 24 (35.3 %) were ESBL negative on confirmation (Table 3). Among the 44 ESBL positive isolates, the majority consist of *E. coli* (77.27 %). Of the total primary screened *E. coli* 72.3 % were ESBL positive.

**Table 3:** ESBL production by various isolates

Organisms	No. of suspected ESBL positive isolates	ESBL production confirmatory test			
		Positive	%	Negative	%
*Acinetobacter* spp.	3	1	33.3	2	66.7
*Citrobacter koseri*	1	1	100	0	0
*Citrobacter freundii*	1	1	100	0	0
*E. coli*	47	34	72.3	13	27.7
*Edwardsiella* spp.	1	1	100	0	0
*Enterobacter* spp.	2	2	100	0	0
*Kl. pneumoniae*	5	2	40	3	60
*Ps. aeruginosa*	8	2	25	6	75
Total	68	44	64.7	24	35.3

### Confirmation of ESBL production using 3 different cephalosporins and respective clavulanic acid combination disks

Among the 68 suspected ESBL positive isolates that were subjected to ESBL confirmation test using three different combination disks, the ceftazidime/ceftazidime+ clavulanate detected 44 ESBL positive isolates whereas cefpodoxime/cefpodoxime+ clavulanate and cefotaxime/cefotaxime+ clavulanate detected 38 and 36 ESBL positive isolates respectively (Table 4). Ceftazidime/clavulanate was found relatively suitable in detection of ESBL positive isolates and was statistically significant (P < 0.01).

**Table 4:** ESBL production detected by 3 different combination disks

Combination disks used	Criteria for confirmation	No. of suspected ESBL producers	No. of confirmed ESBL positive cases	Total confirmed ESBL positive cases	ESBL negative after confirmation
CPD, CPD+CV	Increase in zone size of ≥5 mm with ≥1 combination disks	68	38	44	24
CTX, CTX+CV			36		
CAZ, CAZ+CV			44		

*CPD* cefpodoxime, *CAZ* ceftazidime, *CTX* cefotaxime, *CV* clavulanate

## Discussion

Similar to our study the studies carried out by Tankhiwale et al., Begum et al. and Baral et al. showed a low percentage of growth positivity [8–10]. The reason behind the low growth rate observed in this study might be due to the prior use of antibiotics, infection due to slow growing organisms or due to those organisms that were not able to grow on the routine media we used. Our findings were in accordance with the findings of Awasthi et al. [11] who found 95.92 % of the urinary isolates to be gram negative bacteria and 4.08 % of the isolates to be gram positive bacteria. Higher prevalence of *E. coli* seen in our study also resembled the results of the studies done by other researchers [10, 12–14].

Our finding regarding the susceptibility of the gram negative bacteria toward nitrofurantoin resembled the findings reported by Tankhiwale et al. and Baral et al. [8, 10]. Nitrofurantoin should be considered as drug of choice for acute, uncomplicated UTI due to its specificity toward urinary tract and low level of in vitro resistance shown by the bacteria [15]. Nitrofurantoin is not generally prescribed in UTI caused by *Proteus* spp. as this urease producing organism render urine alkaline and thus decreasing the potency of the drug in vivo [16]. Among the antibiotics evaluated secondarily, highest numbers of the gram negative isolates were found to be susceptible to imipenem followed by amikacin. Higher resistance to penicillins seen in our study might be due to the production of penicillin destroying enzymes (β-lactamases) by the bacteria.

In our study, the rate of ESBL production among the uropathogens was similar to the rates of ESBL production reported by other studies [2, 10]. In our study three different cephalosporins combination with clavulanic acid were used for confirmation of ESBL production and maximum ESBL production was detected by CAZ/CAZ+CV combination which correlated with another study [17].

## Limitation of the study

Since this study was conducted in the low income country, where the availability of the advanced laboratory is not easy, we could not use molecular techniques to confirm our findings.

## Conclusion

Among the three different combination disks, CAZ/CAZ+CV was found to be the best combination in confirmation of ESBL production. Routine ESBL production testing for uropathogens along with conventional antibiogram would be useful for early proper management of all cases of UTIs.

## Abbreviations

UTI: urinary tract infection
ESBL: extended spectrum β-lactamase
